# Weaning Stress Aggravates Defense Response and the Burden of Protein Metabolism in Low-Birth-Weight Piglets

**DOI:** 10.3390/ani15101369

**Published:** 2025-05-09

**Authors:** Peipei Wang, Jinwei Zhang, Yihang Tian, Bing Yu, Jun He, Jie Yu, Ping Zheng

**Affiliations:** 1 Institute of Animal Nutrition, Sichuan Agricultural University, Chengdu 611130, China; gfawpp@163.com (P.W.); 18227585873@163.com (Y.T.); ybingtian@163.com (B.Y.); hejun8067@163.com (J.H.); jerryyujie@163.com (J.Y.); 2 Key Laboratory of Animal Disease-Resistant Nutrition, Chengdu 611130, China; 3 Department of Quality Management and Inspection & Quarantine, Yibin University, Yibin 644000, China; zhangjinwei@yibinu.edu.cn

**Keywords:** low birth weight, piglets, weaning stress, intestinal damage

## Abstract

The effect of low birth weight (LBW) on piglet development has been widely demonstrated. However, the reasons for the significant decline in the growth performance of LBW piglets after weaning remain poorly understood. This study aimed to investigate the underlying mechanisms of this phenomenon. At 21 days of age, 24 normal-birth-weight (NBW) piglets and 24 LBW piglets were selected and divided into four groups—NBW control, NBW weaning, LBW control, and LBW weaning—with 12 replicates per group (1 piglet per replicate). Control groups were euthanized on the same day, while weaning groups were weaned and sampled 3 days later. The results showed that the body weight of NBW piglets increased, whereas that of LBW piglets decreased, after 3 days of weaning. Compared with NBW piglets, LBW piglets exhibited higher serum cortisol concentrations and lower villus height (*p* < 0.05). Weaning stress significantly increased serum cortisol and C-reactive protein concentrations in NBW piglets (*p* < 0.05), while no significant changes were observed in LBW piglets. However, weaning stress significantly increased serum blood urea nitrogen (BUN) concentrations (*p* < 0.05) in LBW piglets but not in NBW piglets. Additionally, weaning stress reduced the mRNA expressions of *Occludin*, *Claudin-1*, and *Claudin-2* in the jejunum of NBW piglets (*p* < 0.05), as well as *Occludin* in the jejunum of LBW piglets (*p* < 0.05). Furthermore, weaning stress reduced the mRNA expressions of *IL-6*, *TLR9*, *MyD88*, *TRIF* and *p65 NF-κB* in the jejunum of NBW piglets (*p* < 0.05). In LBW piglets, weaning stress decreased the mRNA expressions of *IL-2*, *TNF-α*, *NLRP3*, *TLR9*, and *NOD2* (*p* < 0.05). In conclusion, compared to NBW piglets, LBW piglets are more susceptible to weaning stress-induced protein metabolic disorders and intestinal barrier dysfunction, ultimately leading to impaired immune function and reduced growth performance. The results underscore the importance of tailored management strategies for piglets based on birth weight to mitigate weaning stress impacts.

## Simple Summary

The reasons for the significant decline in growth performance of low-birth-weight piglets after weaning remain incompletely characterized. This study aimed to investigate the underlying mechanism of this phenomenon. The results demonstrated that the stress resistance capacity of low-birth-weight piglets reached its peak at weaning. Furthermore, weaning stress increased the protein metabolic burden in low-birth-weight piglets, leading to metabolic dysregulation and further impairment of intestinal barrier function. This study suggests that low-birth-weight piglets exhibit impaired weaning adaptability, and strategies such as delaying weaning and reducing dietary protein levels may help alleviate this condition.

## 1. Introduction

With the development of the livestock industry and the genetic selection for high reproductive performance, the average litter size of sows has significantly increased. Elevated litter sizes exert increased demands on sow uterine capacity [1,2]. Insufficient maternal provision to meet fetal developmental requirements may induce intrauterine growth restriction (IUGR), consequently resulting in a significantly higher proportion of low-birth-weight (LBW) piglets within the affected litter [3,4,5,6,7]. LBW piglets, typically defined as those with a birth weight below 1.0 kg, have a higher mortality rate before weaning [8,9]. This condition has led to substantial economic losses in the pig industry.

The small intestine plays a pivotal role in nutrient metabolism, serving as the primary site for digestion and absorption, as well as acting as a critical defense barrier. It is generally believed that improving animal production performance is closely related to improving intestinal function [10]. Currently, the impact of LBW on the intestinal health of piglets has received extensive attention. Previous studies have indicated that the growth retardation in LBW piglets is partly caused by impaired small intestine function [11]. Subsequent studies have demonstrated that intestinal dysfunction in LBW piglets is associated with impaired intestinal morphology and physical barriers, as well as increased intestinal permeability [12,13,14]. Greater villus height and a higher villus height-to-crypt depth (V/C) ratio indicate more advanced development of the digestive tract in piglets, enhancing their growth performance and digestive capacity [15]. In addition, the intestinal tract is recognized as the largest immune organ in piglets, containing a higher number of immune cells and tissues, which are significant for maintaining the intestinal health of piglets. Recent studies have shown that elevated levels of pro-inflammatory cytokines in the intestine and reduced expression of immune-related genes might be partly responsible for intestinal dysfunction in LBW piglets [16,17]. The impaired intestinal function of LBW piglets reduces nutrient absorption capacity [18]. This leads to excess nutrients reaching the hindgut, promoting the overgrowth of harmful microorganisms and disrupting microbial homeostasis [19,20]. Since the intestinal immune system maintains microbial homeostasis under normal conditions [21], this microbial imbalance indicates further deterioration of intestinal immune function in LBW piglets.

Weaning stress is one of the greatest challenges that piglets encounter. Many studies have reported the effects of weaning on normal-birth-weight (NBW) piglets, including reduced feed intake, impaired intestinal function, and weakened immune function [22,23]. In contrast, research on the impact of weaning stress on LBW piglets is limited. Although all piglets have difficulty adapting to a post-weaning diet, this is particularly prominent in LBW piglets. A comparative analysis of feed intake, growth rate, and feed conversion rate between NBW and LBW piglets after weaning demonstrated that LBW piglets were prone to negative growth after weaning [24,25]. Michiels et al. found that LBW piglets suffer from delayed gastrointestinal maturation after weaning stress [26], manifested by lower small intestine weight-to-length ratios, reduced oxidation capacity, and lower digestive capacity. Therefore, the effects of weaning stress may vary depending on the birth weight of the piglets, with lighter piglets inherently more susceptible to weaning stress than heavier ones. However, the reasons for the different effects of weaning stress on LBW piglets and NBW piglets remain unclear. Therefore, this study aims to explore why LBW piglets perform poorly after weaning and how weaning stress exacerbates the adverse effects of LBW in piglets.

## 2. Materials and Methods

### 2.1. Experimental Design and Animal Management

The study protocol received approval from the Animal Experimental Committee of Sichuan Agricultural University (SAU20200321). The study was conducted at the Animal Experiment Center of Sichuan Tieqi Breeding Co., Ltd., located in Mianyang, China.

LBW piglets are defined as piglets with birth weights below 1.0 kg, while NBW piglets are defined as those whose birth weights within the mean range of the entire litter (within ± 1.0 SEM), as detailed in our previous study [27]. A total of 30 NBW and 30 LBW piglets were selected from 30 sows with similar parity (3rd–5th parity). On day 1, each piglet was ear-tagged and subjected to routine procedures, including tail docking and tooth clipping. To minimize inter-group variation caused by differences between litters, 24 NBW piglets (6.16 ± 0.03 kg) and 24 LBW piglets (3.65 ± 0.07 kg) were selected. The piglets were grouped based on similar average body weights, with litters randomized and a sex ratio of 1:1. The groups included the NBW control group, NBW weaning group, LBW control group, and LBW weaning group, with 12 replicates per group, each consisting of 1 piglet. All sows and piglets used in the experiment were healthy, vaccinated, and free of infectious diseases. The control groups were sampled at 21 days old, while the weaning groups were sampled 3 days after weaning. During the experiment, the experimental piglets were uniformly fed with creep feed (Supplementary Table S1) as supplementary feeding treatment.

### 2.2. Sample Collection

Blood samples were collected from the anterior vena cava into non-heparinized vacuum tubes. After centrifugation (3500× *g*, 10 min, 4 °C), the serum was separated and stored at −20 °C for subsequent analysis of serum parameters. Following blood collection, piglets were euthanized with a lethal dose of sodium pentobarbital (200 mg/kg of BW) following procedures outlined in a previous study [28], and the abdomens were immediately incised to collect intestinal samples. The entire small intestine was excised and divided into three segments: duodenum, jejunum, and ileum, as previously described by Zheng et al. [29]. Approximately 2 cm segments of the proximal jejunum were immediately isolated, and preserved in 4% paraformaldehyde solution after being rinsed, for histological analysis. Subsequently, 10 cm segments of jejunum were emptied, rinsed, and placed on an ice-cold surface. The jejunal mucosa was scraped using glass microscope slide, and the samples were frozen in liquid nitrogen and stored at −80 °C for further analysis. Additionally, digesta from the middle of cecum and colon were collected and stored at −80 °C for microbial and metabolite analysis.

### 2.3. Serum Physiochemical Parameters

Serum cortisol (COR), blood urea nitrogen (BUN), and C-reactive protein (CRP) concentrations were measured using enzymatic colorimetric methods, following the instructions provided by commercial assay kits (Nanjing Jiancheng Co., Ltd., Nanjing, China).

### 2.4. Determination of Histological Analysis

Jejunum morphology was analyzed according to the method described by Wang et al. [30]. Briefly, samples were fixed in neutral buffered formaldehyde, dehydrated, and embedded in paraffin. Four transverse sections (5 μm) were cut, mounted on slides, and stained with hematoxylin-eosin. Villus height and crypt depth were quantified using an Olympus CK 40 microscope (Olympus Optical Company, Shenzhen, China). A minimum of 10 intact villi and associated crypts from each intestinal segment were measured.

### 2.5. Total RNA Extraction and Real-Time Quantitative PCR

Jejunum mucosal samples (approximately 0.1 g) were homogenized in 1 mL RNAiso Plus reagent (TaKaRa, Dalian, China) to extract total RNA according to the manufacturer’s instructions. RNA concentration and quality were assessed using a Beckman Coulter DU 800 spectrophotometer (Beckman Coulter Inc., Brea, CA, USA). First-strand complementary DNA (cDNA) was synthesized from each sample using the Prime Script^TM^ RT reagent kit (TaKaRa, Dalian, China) following the manufacturer’s protocol.

Specific primers for zonula occludens-1 (*ZO-1*), zonula occludens-2 (*ZO-2*), *Occludin*, *Claudin-1*, *Claudin-2*, interleukin-1β (*IL-1β*), interleukin-2 (*IL-2*), interleukin-6 (*IL-6*), tumor necrosis factor-α (*TNF-α*), interferon-γ (*IFN-γ*), NOD-like receptor thermal protein domain associated protein 3 (*NLRP3*), interleukin-18 (*IL-18*), *Caspase-1*, Toll-like receptor 4 (*TLR4*), Toll-like receptor 9 (*TLR9*), nucleotide binding oligomerization domain (NOD)-like receptor 1 (*NOD1*), NOD-like receptor 2 (*NOD2*), myeloid differentiation factor 88 (*MyD88*), tumor necrosis factor receptor-associated factor 6 (*TRAF6*), TIR domain–containing adaptoc–inducing IFN-β (*TRIF*), p38 mitogen-activated protein kinase (*p38 MAPK*), interferon regulatory factor 3 (*IRF3*), and p65 nuclear factor kappa B (*p65 NF-κB*) were designed and purchased from Invitrogen (Shanghai, China). Primer sequences are listed in Table 1.

Real-time PCR reactions were conducted using a CFX96TM Real-Time PCR detection system (Bio-Rad Laboratories, Inc., Hercules, CA, USA) with a SYBR Green PCR reagent kit (TaKaRa, Dalian, China). The amplification was carried out with the following cycling conditions: an initial denaturation at 95 °C for 30 s, followed by 40 cycles of 95 °C for 10 s (denaturation), annealing at the appropriate temperature for 25 s, and a final extension at 72 °C for 5 s. Melting curve analysis was performed after each quantitative PCR to confirm the specificity and purity of the PCR products.

The β-actin gene was employed as the reference gene to normalize cDNA loading. To calculate amplification efficiencies, a 10-fold serial dilution was performed to generate standard curves for both the target and reference genes, using six different concentrations. After verification, the primers exhibited an amplification efficiency of approximately 100%. The results were calculated using the 2^−ΔΔCt^ method [31]. Analysis of each standard and sample was run in triplicate simultaneously on the same PCR plate, and the average of each triplicate value expressed as the number of copies was used for subsequent statistical analysis.

### 2.6. DNA Extraction and Quantification of Intestinal Microflora

Microbial genomic DNA was extracted from approximately 0.2 g of digesta samples using the E.Z.N.A stool DNA kit (Omega Bio-tek, Doraville, GA, USA) according to the manufacturer’s instructions. Primers and probes (Table 2) targeting total bacteria, *Escherichia coli* (*E. coli*), *Lactobacillus*, and *Bacillus* were designed based on the studies by Fierer et al. and Qi et al. [32,33], and were commercially synthesized by Invitrogen (Shanghai, China). Quantitative real-time PCR was conducted using a CFX96 Real-Time PCR Detection System (Bio-Rad Laboratories, Inc., Hercules, CA, USA).

To determine the total bacteria, the thermal cycling conditions were as follows: an initial pre-denaturation at 95 °C for 10 s, followed by 40 cycles of denaturation at 95 °C for 5 s, annealing at 60 °C for 25 s, and extension at 72 °C for 60 s.

Real-time PCR for the quantification of *Lactobacillus*, *E. coli*, *Bifidobacterium*, and *Bacillus* was performed in a 20 μL reaction volume, consisting of 1 μL probe enhancer solution, 0.3 μL probe, 1 μL forward primer, 1 μL reverse primer, 8 μL RealMasterMix (Tiangen, Beijing, China), 1 μL template DNA, and 7.7 μL ultrapure water. The PCR protocol included an initial denaturation at 95 °C for 10 s, followed by 50 cycles consisting of 5 s at 95 °C, 25 s at the annealing temperature, and 60 s at 72 °C. Copies per sample were calculated using the threshold cycle (CT) values and standard curve based on previous work by Xiang et al. [34].

### 2.7. Statistical Analysis

The data were analyzed using Tukey’s multiple comparisons for the 2 × 2 factorial experimental design, using the General Linear Model (GLM) procedure in SPSS statistical software (Ver.20.0 for Windows, SPSS; IBM, Armonk, NY, USA) with pen as the experimental unit (*n* = 12). The results are presented as means ± standard error of the mean (SEM). Statistical significance was determined with an α-level of 0.05, where *p* < 0.05 indicated a significant difference and 0.05 ≤ *p* < 0.10 indicated a tendency.

## 3. Results

### 3.1. Body Weight of Piglets

Compared with NBW piglets, LBW piglets had lower (*p* < 0.05) body weight. After 3 days of weaning, the weight of NBW piglets increased by 210 g, while LBW piglets’ body weight decreased by 100 g (Table 3).

### 3.2. Physicochemical Parameters in Serum

**Figure 1:**
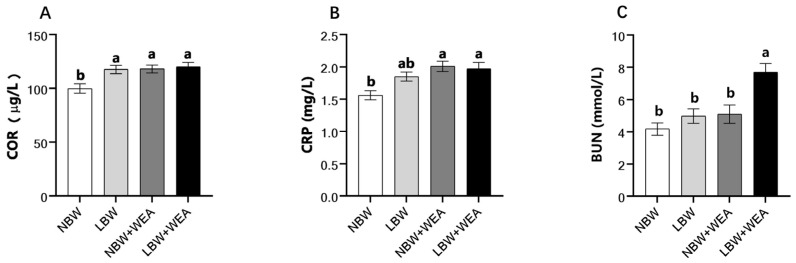
Effects of birth weight and weaning on serum parameters of piglets. (**A**) The concentration of serum cortisol (COR). (**B**) The concentration of serum C-reactive protein (CRP). (**C**) The concentration of blood urea nitrogen (BUN).; WEA = weaning groups; NBW = normal birth weight; LBW = low birth weight. The results are shown as means ± SEM represented by vertical bars (*n* = 12). ^a,b^ Means values with different letters on vertical bars indicate significant differences (*p* < 0.05).

Figure 1 shows the serum physicochemical parameters in NBW and LBW piglets. Compared with NBW piglets, LBW piglets had higher (*p* < 0.05) concentrations of serum COR. Weaning stress increased serum COR and CRP concentrations in NBW piglets (*p* < 0.05) but had no significant effect on LBW piglets (*p* > 0.05). Weaning stress increased BUN concentrations in LBW piglets (*p* < 0.05) but had no significant impact on NBW piglets (*p* > 0.05). However, the concentrations of BUN of LBW weaning piglets were higher (*p* < 0.05) than that of NBW weaning piglets.

### 3.3. Intestinal Morphology

The morphological data of the jejunum are summarized in Table 4. Compared with NBW piglets, LBW piglets had lower (*p* < 0.05) villus height. Weaning stress significantly reduced both jejunal villus height and villus height/crypt depth in NBW piglets (*p* < 0.05), with a concurrent significant reduction in villus height/crypt depth observed in LBW piglets (*p* < 0.05).

### 3.4. Gene Expression of Tight Junction Proteins

**Figure 2:**
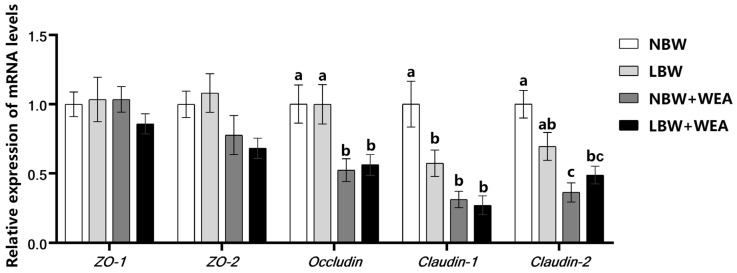
Effects of birth weight and weaning on expression of tight junction protein genes in jejunum of piglets. WEA = weaning groups; NBW = normal birth weight; LBW = low birth weight; *ZO-1* = zonula occludens-1; *ZO-2* = zonula occludens-2. The results are shown as means ± SEM represented by vertical bars (*n* = 12). ^a,b,c^ Means values with different letters on vertical bars indicate significant differences (*p* < 0.05).

As shown in Figure 2, it was found that compared with NBW piglets, LBW piglets had lower (*p* < 0.05) mRNA expression of *Claudin-1* in the jejunum. Weaning stress significantly decreased the mRNA expression of *Occludin*, *Claudin-1*, and *Claudin-2* in the jejunum of NBW piglets (*p* < 0.05), and also reduced jejunum *Occludin* mRNA expression of LBW piglets (*p* < 0.05). There was a significant (*p* < 0.05) interaction effect of birth weight and weaning stress on the mRNA expression of *Claudin-2*.

### 3.5. Gene Expression of Inflammation-Related Genes

**Figure 3:**
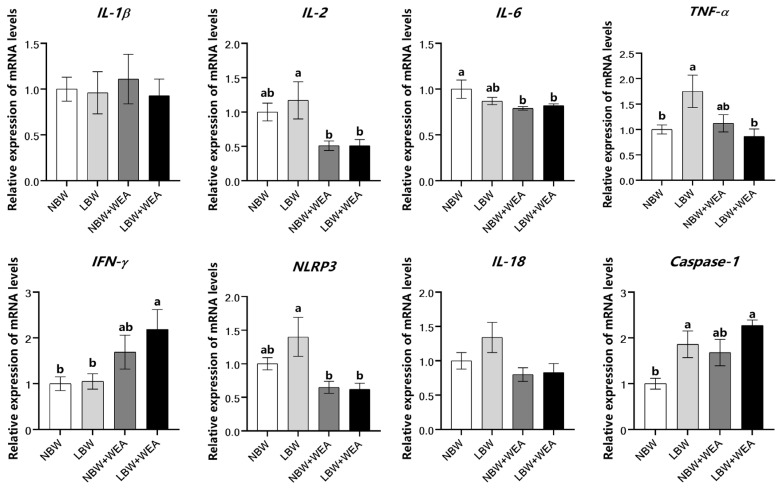
Effects of birth weight and weaning on expression of inflammation-related genes in jejunum of piglets.; WEA = weaning groups; NBW = normal birth weight; LBW = low birth weight; *IL-1β* = interleukin-1β; *IL-2* = interleukin-2; *IL-6* = interleukin-6; *TNF-α* = tumor necrosis factor α; *IFN-γ* = Interferon-γ; *NLRP3* = NOD-like receptor thermal protein domain associated protein 3; *IL-18* = interleukin-18. The results are shown as means ± SEM represented by vertical bars (*n* = 12). ^a,b^ Means values with different letters on vertical bars indicate significant differences (*p* < 0.05).

As shown in Figure 3, the results showed that compared with NBW piglets, LBW piglets had higher (*p* < 0.05) mRNA expression of *Caspase-1* and *TNF-α* in the jejunum. Weaning stress decreased (*p* < 0.05) mRNA expressions of *IL-6* in the jejunum of NBW piglets. Weaning stress increased (*p* < 0.05) mRNA expressions of *IFN-γ*, and decreased (*p* < 0.05) mRNA expressions of *IL-2*, *TNF-α*, and *NLRP3* in the jejunum of LBW piglets. There was a significant interaction (*p* < 0.05) effect of birth weight and weaning stress on mRNA expressions of *TNF-α*.

### 3.6. Gene Expression of Immune-Related Genes

**Figure 4:**
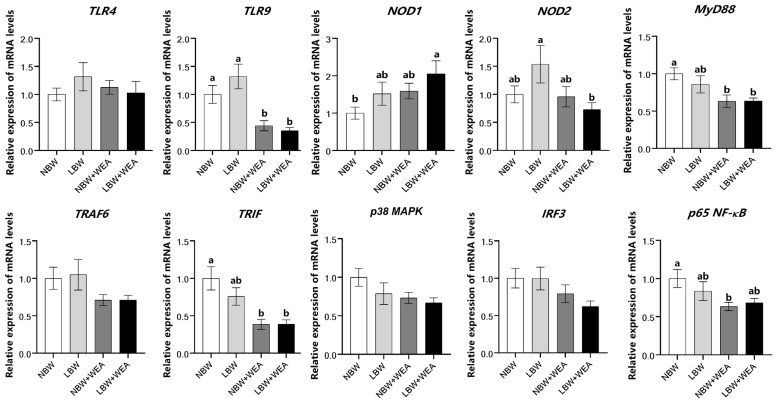
Effects of birth weight and weaning on expression of immune-related genes in jejunum of piglets.; WEA = weaning groups; NBW = normal birth weight; LBW = low birth weight; *TLR4* = Toll-like receptors 4; *TLR9* = Toll-like receptors 9; *NOD* = nucleotide-binding oligomerization domain protein; *NOD1* = NOD-like receptor 1; *NOD2* = NOD-like receptor 2; *MyD88* = myeloid differentiation factor 88; *TRAF6* = tumor necrosis factor receptor-associated factor 6; *TRIF* = TIR domain-containing adaptor inducing IFN-β; *p38 MAPK* = p38 mitogen-activated protein kinase; *IRF3* = interferon regulatory factor 3; *p65 NF-κB* = p65 nuclear factor κB. The results are shown as means ± SEM represented by vertical bars (*n* = 12). ^a,b^ Means values with different letters on vertical bars indicate significant differences (*p* < 0.05).

As shown in Figure 4, the results showed that birth weight had no significant effect on the expression of immune-related genes in the jejunum of piglets. After weaning, the mRNA expressions of *TLR9*, *MyD88*, *TRIF*, and *p65 NF-κB* in the jejunum of NBW piglets were significantly decreased (*p* < 0.05). The mRNA expressions of *TLR9* and *NOD2* in the jejunum of LBW piglets were significantly decreased (*p* < 0.05).

### 3.7. Intestinal Microbiota

As shown in Table 5, the results showed birth weight had a significant effect (*p* < 0.05) on the abundance of *E. coli* in the cecal digesta of piglets. Compared with pre-weaning levels, post-weaning LBW piglets showed a significant increase in *E. coli* abundance in cecal digesta (*p* < 0.05). Furthermore, the abundance of *E. coli* in the cecal digesta of LBW weaned piglets was higher (*p* < 0.05) than that of NBW weaned piglets.

## 4. Discussion

In order to reduce the sow production cycle and improve the breeding efficiency, early weaning is usually adopted in the pig industry. Typically, early weaning occurs between 21 and 28 days of age [35]. Early weaning exposes piglets to a variety of health challenges, including maternal and littermate separations, changes in diet, intestinal damage, digestive disorders, energy metabolism disturbances, and immune dysfunction [23,36,37]. These challenges are particularly pronounced in LBW piglets, which exhibit a higher risk of infection and a lower overall survival rate compared to NBW piglets [38,39]. Our results demonstrated that the weight of NBW piglets increased after weaning, while the weight of LBW piglets decreased. This disparity in post-weaning growth performance between LBW and NBW piglets may be attributed to their distinct physiological and molecular responses to weaning stress. The potential reasons for the huge difference in growth between NBW and LBW piglets after weaning are as follows:

First, the anti-stress ability of LBW piglets peaks at weaning. Previous studies have revealed that weaning stress can change the endocrine system of piglets, thereby affecting the function of the hypothalamic–pituitary–adrenal axis, increasing serum COR concentrations [40,41,42]. Consequently, changes in cortisol levels can serve as an indicator for diagnosing stress in piglets [43]. Similarly, CRP, an acute-phase serum protein, plays a role in innate and adaptive immunity [44]. Serum CRP levels can reflect the presence of inflammation and infection in pigs [45]. According to Wang et al., piglets with post-weaning diarrhea exhibit higher stress levels, as indicated by elevated concentrations of CRP and COR, compared to healthy piglets [46]. Our study revealed that the serum COR concentrations of LBW piglets were significantly higher than those of NBW piglets before weaning. This result indicates that LBW piglets are already under stress before weaning. The concentrations of COR and CRP in NBW piglet serum increased significantly 3 days after weaning. This indicates that NBW piglets were subjected to weaning stress, consistent with a previous study [47]. However, the concentrations of COR and CRP in LBW piglets did not significantly increase after weaning, likely because their stress tolerance had already peaked prior to weaning and they were in a state of immune exhaustion, rendering them incapable of coping with additional weaning stress.

Second, weaning stress can further increase the protein metabolism load of LBW piglets, resulting in abnormal protein metabolism. BUN is a serum by-product of protein catabolism, and its level reflects the body’s protein metabolism and amino acid utilization efficiency [48,49]. This research showed that BUN concentrations were higher in LBW piglets than in NBW piglets. Moreover, weaning stress significantly increased BUN concentrations in LBW piglets. Increased BUN concentrations are associated with decreased protein synthesis and increased protein catabolism [50]. Therefore, it can be speculated that LBW increases nitrogen metabolism in piglets, which negatively affects protein deposition. Furthermore, weaning stress can increase BUN concentrations in piglets [51,52]. Combined with the post-weaning weight loss observed in LBW piglets, these findings suggest that weaning stress impairs dietary protein utilization. Excessive dietary protein will further aggravate the metabolic burden, suggesting that LBW piglets should not be fed high-protein diets.

Third, weaning stress leads to a further weakening in the intestinal functions of LBW piglets. Our study demonstrated significantly reduced jejunum villus height in LBW piglets, indicating delayed intestinal morphological development that likely compromises nutrient absorption capacity. Normal epithelial barrier function primarily relies on tight junctions (TJs), which serve as both ion channels and barriers against harmful molecules [53]. After weaning, the mRNA expression levels of tight junction proteins, including *Occludin*, *Claudin-1*, and *Claudin-2*, were significantly downregulated in NBW piglets, while *Occludin* expression levels were also decreased in LBW piglets. Due to the strong correlation between mRNA expression and tight junction protein levels, the observed downregulation of these markers suggests that weaning stress impairs intestinal barrier function in piglets [54,55]. Previous studies found that the expression of tight junction proteins in the jejunum of NBW and LBW piglets decreased significantly [56,57]. However, the damaged proteins of NBW and LBW piglets were different, which may have different mechanisms. Furthermore, the gut microbiota is often referred to as an “additional forgotten organ,” playing a crucial role in maintaining intestinal barrier integrity in animals. Weaning stress compromises intestinal barrier function by reducing the expression of tight junction proteins, which makes the intestinal tract of piglets more vulnerable to the invasion of pathogenic microorganisms such as *E. coli* [58,59,60,61]. Additionally, during the early weaning phase, the immature digestive system of piglets limits their capacity to fully digest and absorb dietary nutrients. This incomplete digestion provides a substrate for the overgrowth of pathogenic bacteria, which further disrupts the intestinal microbial barrier and exacerbates intestinal dysfunction [17,62]. Studies have found that pathogenic *Escherichia coli* is more likely to colonize the intestinal tract of piglets, thereby triggering the occurrence of intestinal immune and inflammatory responses and reducing intestinal health [63,64]. In this study, the abundance of *E. coli* in cecal digesta of LBW piglets increased significantly after weaning compared to pre-weaning levels, and the abundance of *E. coli* in cecal digesta of LBW weaned piglets was significantly higher than that of NBW weaned piglets. Thus, it is hypothesized that weaning stress in LBW piglets disrupts intestinal barrier function and gut microbiota balance, particularly *E. coli* abundance, further impairing intestinal development. Compared to NBW piglets, LBW piglets show more severe responses to weaning stress, indicating their greater vulnerability.

Meanwhile, weaning stress leads to an imbalance in inflammation and immune response in LBW piglets. *Caspase-1*, a best-characterized inflammatory caspase, serves as the central effector protein of the inflammasome and plays a critical role in mediating inflammatory responses [65]. In the present study, we observed elevated expression of *Caspase-1* in LBW piglets before weaning, which may indicate an activated inflammasome pathway and a potential state of low-grade intestinal inflammation [66]. Following weaning stress, distinct patterns of inflammatory responses were observed between NBW and LBW piglets. In NBW piglets, weaning led to a reduction in the expression of *IL-6*, suggesting a potential attenuation of pro-inflammatory signaling. In contrast, LBW piglets exhibited increased expression of *IFN-γ* alongside inhibition of the *TNF-α* and *NLRP3* pathways, as evidenced by the downregulation of *IL-2*, *TNF-α* and *NLRP3*. These findings suggest that LBW piglets may experience immune suppression or immune depletion after weaning, whereas NBW piglets may mount a more localized or transient inflammatory response. Further analysis revealed that LBW piglets may mitigate excessive inflammation through the inhibition of *TNF-α*, albeit at the cost of compromised immune defense capabilities. Additionally, weaning stress differentially impacted immune signaling pathways in NBW and LBW piglets. In NBW piglets, the *TLR9/MyD8*8/*NF-κB* pathway was suppressed, while *NOD1* expression exhibited an upregulation trend, suggesting a potential shift toward intracellular pathogen recognition [67]. The inhibition of the *TLR9/NOD2* pathway in LBW piglets may impair innate immune responses, thereby increasing susceptibility to infections [68,69]. These results demonstrate the divergent immune adaptations of NBW and LBW piglets to weaning stress and underscore the need for targeted nutritional or management strategies to support LBW piglets during this critical period. In summary, our findings indicate that LBW adversely affects intestinal structure, immune function, and inflammatory status in piglets, and weaning stress exacerbates these adverse effects. LBW impairs the development of the small intestine, leading to reduced digestion and absorption of nutrients [70]. Previous research by De Vos et al. demonstrated that milk-derived bioactive compounds promote gastrointestinal development, suggesting that supplementing LBW piglets with milk replacers could enhance gastrointestinal maturation and function [7]. Furthermore, dietary supplementation with L-arginine has been shown to improve intestinal development and support growth performance in LBW piglets [27]. Therefore, to address the challenges associated with LBW, we recommend providing LBW piglets with milk replacers or supplementing their diet with L-arginine.

## 5. Conclusions

This study confirmed that LBW negatively impacts intestinal mucosal structure, immunity, and inflammatory status in piglets. Meanwhile, the anti-stress ability of LBW piglets peaked at weaning. Furthermore, weaning stress increases the protein metabolism load of LBW piglets, leading to metabolic abnormalities, and further weakened intestinal function. These results contribute to a better understanding of the reasons for the poor growth performance of LBW piglets after weaning. Based on our findings, the intestinal barrier dysfunction of LBW piglets is particularly pronounced at weaning, concomitant with increased metabolic burden. Therefore, it is necessary to improve LBW piglets’ weaning adaptation either through delayed weaning or by reducing dietary protein levels to support their healthy development.

## Figures and Tables

**Table 1:** Sequence of primers used for the real-time quantitative PCR analysis.

Genes ^1^	Primer Sequences (5′-3′) ^2^	Size (bp)	A_T_^3^, °C
*ZO-1*	F: CAGCCCCCGTACATGGAGA	105	59.7
	R: GCGCAGACGGTGTTCATAGTT		
*ZO-2*	F: ATTCGGACCCATAGCAGACATAG	100	59.7
	R: GCGTCTCTTGGTTCTGTTTTAGC		
*Occludin*	F: CAGGTGCACCCTCCAGATTG	110	59.7
	R: GGACTTTCAAGAGGCCTGGAT		
*Claudin-1*	F: GCCACAGCAAGGTATGGTAAC	140	59.7
	R: AGTAGGGCACCTCCCAGAAG		
*Claudin-2*	F: CATCGGCAGCAGCATTATC	95	59.7
	R: ACACTTTGCACTGCATCTGG		
*IL-1β*	F: CAGCTGCAAATCTCTCACCA	112	59.7
	R: TCTTCATCGGCTTCTCCACT		
*IL-2*	F: TGCACTAACCCTTGCACTCA	83	59.7
	R: GCAATGGCTCCAGTTGTTTCT		
*IL-6*	F: TTCACCTCTCCGGACAAAAC	122	59.7
	R: TCTGCCAGTACCTCCTTGCT		
*TNF-α*	F: CGTGAAGCTGAAAGACAACCAG	121	59.7
	R: GATGGTGTGAGTGAGGAAAACG		
*IFN-γ*	F: ACCAGGCCATTCAAAGGAGC	90	59.7
	R: CGAAGTCATTCAGTTTCCCAGAG		
*NLRP3*	F: GGAGGAGGAGGAAGAGGAGATA	147	59.7
	R: AGGACTGAGAAGATGCCACTAC		
*IL-18*	F: AGTAACCATCTCTGTGCAGTGT	155	59.7
	R: TCTTATCATCATGTCCAGGAAC		
*Caspase-1*	F: GAAGGAGAAGAGGAGGCTGTT	268	59.7
	R: AGATTGTGAACCTGTGGAGAGT		
*TLR4*	F: TTACAGAAGCTGGTTGCCGT	152	65.0
	R: TCCAGGTTGGGCAGGTTAGA		
*TLR9*	F: AATCCAGTCGGAGATGTTTGCT	79	59.7
	R: GACCGCCTGGGAGATGCT		
*NOD1*	F: TCAACACCGATCCAGTGAGC	237	59.7
	R: TGAAAATGGTCTCGCCCTCC		
*NOD2*	F: GTGCCTCCCCTCTAGACTCA	191	59.7
	R: ACGAACCAGGAAGCCAAGAG		
*MyD88*	F: CCATTCGAGATGACCCCCTG	183	59.7
	R: TAGCAATGGACCAGACGCAG		
*TRAF6*	F: GCTGCATCTATGGCATTTGAAG	70	59.7
	R: CCACAGATAACATTTGCCAAAGG		
*P38 MAPK*	F: AGTTGAAGCTCATTTTAAGACTCGT	117	59.7
	R: AGTTCATCTTCGGCATCTGGG		
*TRIF*	F: CAAGTGGAGGAAGGAACAGG	139	59.7
	R: CAACTGCGTCTGGTAGGACA		
*IRF3*	F: GCTACACCCTCTGGTTCTGC	95	59.7
	R: GAGACACATGGGGACAACCT		
*p65 NF-* *κ* *B*	F: GTGTGTAAAGAAGCGGGACCT	139	59.7
	R: CACTGTCACCTGGAAGCAGAG		
*β-actin*	F: TCTGGCACCACACCTTCT	114	59.7
	R: TGATCTGGGTCATCTTCTCAC		

^1^ *ZO-1* = zonula occludens-1; *ZO-2* = zonula occludens-2; *IL-1β* = interleukin-1β; *IL-2* = interleukin-2; *IL-6* = interleukin-6; *TNF-α* = tumor necrosis factor α; *IFN-γ* = Interferon-γ; *NLRP3* = NOD-like receptor thermal protein domain associated protein 3; *IL-18* = interleukin-18; *TLR4* = Toll-like receptors 4; *TLR9* = Toll-like receptors 9; *NOD* = nucleotide-binding oligomerization domain protein; *NOD1* = NOD-like receptor 1; *NOD2* = NOD-like receptor 2; *MyD88* = myeloid differentiation factor 88; *TRAF6* = tumor necrosis factor receptor-associated factor 6; *p38 MAPK* = p38 mitogen-activated protein kinase; *TRIF* = TIR domain-containing adaptor inducing IFN-β; *IRF3*= interferon regulatory factor 3; *p65 NF-κB* = p65 nuclear factor κB. ^2^ F = forward primer; R = reverse primer. ^3^ A_T_ = annealing temperature.

**Table 2:** Sequence of primers and probes used for the real-time quantitative PCR analysis of microbial populations.

Primer	Nucleotide Sequence (5′-3′) ^1^	Product Size, bp	A_T_^2^, °C
Total bacteria	F: ACTCCTACGGGAGGCAGCAG R: ATTACCGCGGCTGCTGG	200	60
*Escherichia coli*	F: CATGCCGCGTGTATGAAGAA R: CGGGTAACGTCAATGAGCAAA P: AGGTATTAACTTTACTCCCTTCCTC	96	60
*Lactobacillus*	F: ACTCCTACGGGAGGCAGCAG R: CAACAGTTACTCTGACACCCGTTCTTC P: AAGAAGGGTTTCGGCTCGTAAAACTC-TGTT	126	60
*Bacillus*	F: GCAACGAGCGCAACCCTTGA R: TCATCCCCACCTTCCTCCGGT P: CGGTTTGTCACCGGCAGTCACCT	92	60

^1^ F = forward primer; R = reverse primer; P = probe. ^2^ A_T_ = annealing temperature.

**Table 3:** Effects of weaning on body weight of NBW and LBW piglets ^1^.

Items	NBW ^2^	LBW ^3^	*p*-Value
21 d body weight, kg	6.16 ± 0.03	3.65 ± 0.07	0.000
24 d body weight, kg	6.37 ± 0.03	3.55 ± 0.06	0.000
Body weight gain, g	+210	−100	

^1^ Values are means ± SEM of 12 piglets per group. ^2^ NBW = Normal birth weight. ^3^ LBW = Low birth weight.

**Table 4:** Effects of birth weight and weaning on jejunal morphology in piglets ^1^.

Items	CON ^2^		WEA ^3^		*p*-Value		
	NBW ^4^	LBW ^5^	NBW ^4^	LBW ^5^	BW	WEA	BW × WEA
Villus height, μm	276.60 ± 15.55 a	190.23 ± 35.02 b	137.25 ± 17.69 b	127.29 ± 9.84 b	0.038	0.000	0.093
Crypt depth, μm	67.18 ± 5.25 ab	55.81 ± 6.37 b	73.97 ± 2.60 a	66.25 ± 2.74 ab	0.048	0.072	0.692
V/C ^6^	4.18 ± 0.26 a	3.49 ± 0.56 a	1.88 ± 0.27 b	1.95 ± 0.20 b	0.391	0.000	0.294

^1^ Values are means ± SEM of 12 piglets per group. ^2^ CON = control groups. ^3^ WEA = weaning groups. ^4^ NBW = normal birth weight. ^5^ LBW = low birth weight. ^6^ V/C = villus height/crypt depth. ^a,b^ Means values with different letters on vertical bars indicate significant differences (*p* < 0.05).

**Table 5:** Effects of birth weight and weaning on the microbial populations (log cfu/g of wet digesta) in cecal and colonic digesta of piglets, qPCR results ^1^.

Items	CON ^2^		WEA ^3^		*p*-Value		
	NBW ^4^	LBW ^5^	NBW ^4^	LBW ^5^	BW	WEA	BW × WEA
Cecal digesta							
*Total bacteria*	10.85 ± 0.05	10.52 ± 0.17	10.73 ± 0.13	10.67 ± 0.11	0.102	0.903	0.261
*Lactobacillus*	7.38 ± 0.12	7.11 ± 0.20	7.31 ± 0.25	7.01 ± 0.39	0.279	0.745	0.945
*Escherichia coli*	5.67 ± 0.36 ab	5.10 ± 0.47 b	4.39 ± 0.58 b	6.68 ± 0.25 a	0.044	0.726	0.001
*Bacillus*	5.25 ± 0.07	4.96 ± 0.18	5.40 ± 0.09	5.32 ± 0.12	0.140	0.046	0.388
Colonic digesta							
*Total bacteria*	11.19 ± 0.05	11.21 ± 0.05	11.15 ± 0.05	11.23 ± 0.06	0.355	0.893	0.540
*Lactobacillus*	7.76 ± 0.13	7.73 ± 0.15	7.62 ± 0.24	7.93 ± 0.08	0.380	0.853	0.311
*Escherichia coli*	7.70 ± 0.08	6.97 ± 0.18	7.43 ± 0.21	7.48 ± 0.33	0.126	0.592	0.081
*Bacillus*	5.40 ± 0.08	5.46 ± 0.08	5.46 ± 0.05	5.49 ± 0.07	0.510	0.510	0.856

^1^ Values means *n* = 12 for the four groups. ^2^ CON = control groups. ^3^ WEA = weaning groups. ^4^ NBW = normal birth weight. ^5^ LBW = low birth weight. ^a,b^ Means statistical significance (*p* < 0.05) in a row between the two treatments.

## Data Availability

The data generated and analyzed during this study can be obtained from the corresponding author upon reasonable request. The data are not publicly accessible to safeguard privacy and protect intellectual property.

## Supplementary Materials

The following supporting information can be downloaded at: https://www.mdpi.com/article/10.3390/ani15101369/s1, Table S1: Composition and nutrient levels of creep feed.
