# Supplementary material for: Weaning Stress Aggravates Defense Response and the Burden of Protein Metabolism in Low-Birth-Weight Piglets
Source: Animals (Basel). 2025 May 9;15(10):1369. doi: 10.3390/ani15101369 (PMC12108514; doi:10.3390/ani15101369)
Supplement: Supplementary file 1 [file animals-15-01369-s001.zip › animals-3624319-supplementary.pdf]

Supplementary Table S1. Composition and nutrient levels of creep feed (air-dry basis) %.

**Items**	**Content**
**Ingredients**	
Corn	21.13
Expand corn	20.64
Rough rice	10.00
Soy protein concentrate	2.80
Coconut oil	1.00
Soybean oil	1.00
Plasma protein powder	3.00
Whey powder	5.00
Full-fat expand soybean	10.00
Soybean meal	11.00
Fish meal	3.00
Sucrose	2.00
Glucose	3.00
Whole milk powder	2.00
L-Lys · HCl	0.45
DL-Met	0.15
L-Thr	0.13
Choline chloride (50%)	0.10
Limestone	1.00
$CaHPO_4$	0.70
NaCl	0.20
Mineral premix[1)]	0.15
Vitamin premix[2)]	0.05
Multiple acidifier[3)]	0.50
Chlortetracycline	0.50
Chitosan	0.50
Total	100.00
Nutrient levels[4)]	
DE/ (MJ/kg)	14.84
CP	20.03
Ca	0.81
STTD P	0.47
SID Lys	1.42
SID Met	0.44
SID Met + Cys	0.76
SID Thr	0.83
SID Try	0.22

1) Mineral premix provided the following per kg of the diet: Cu (as copper sulfate) 6 mg, Fe (as ferrous sulfate) 100 mg, Mn (as manganese sulfate) 4 mg, Zn (as zinc sulfate) 100 mg, I (as potassium iodide) 0.30 mg, Se (as sodium selenite) 0.35 mg.

2) Vitamin premix provided the following per kg of the diet: VA 9 000 IU, VB_1 1.5 mg, VB_2 4.0 mg, VB_6 3.0 mg, VB_{12} 0.02 mg, VD_3 3 000 IU, VE 20 IU, VK_3 3.0 mg, biotin 0.1 mg.

3) Multiple acidifier mainly included lactic acid, formic acid and citric acid.

4) Nutrients levels were calculated values.
